# 18F-FDG-PET/MRI texture analysis in rectal cancer after neoadjuvant chemoradiotherapy

**DOI:** 10.1097/MNM.0000000000001570

**Published:** 2022-04-26

**Authors:** Giulia Capelli, Cristina Campi, Quoc Riccardo Bao, Francesco Morra, Carmelo Lacognata, Pietro Zucchetta, Diego Cecchin, Salvatore Pucciarelli, Gaya Spolverato, Filippo Crimì

**Affiliations:** a General Surgery 3, Department of Surgical, Oncological and Gastroenterological Sciences, University of Padova, Padova; b Department of Mathematics, University of Genova, Genova; c Institute of Radiology, Department of Medicine, University of Padova; d Radiology Department, Azienda Ospedaliera di Padova; e Nuclear Medicine Unit, Department of Medicine, University of Padova, Padova, Italy

**Keywords:** neoadjuvant chemoradiotherapy, PET/MRI, radiology, rectal cancer, surgery

## Abstract

Reliable markers to predict the response to neoadjuvant chemoradiotherapy (nCRT) in locally advanced rectal cancer (LARC) are lacking. We aimed to assess the ability of 18F-FDG PET/MRI to predict response to nCRT among patients undergoing curative-intent surgery.

**Methods:**

Patients with histological-confirmed LARC who underwent curative-intent surgery following nCRT and restaging with 18F-FDG PET/MRI were included. Statistical correlation between radiomic features extracted in PET, apparent diffusion coefficient (ADC) and T2w images and patients’ histopathologic response to chemoradiotherapy using a multivariable logistic regression model ROC-analysis.

**Results:**

Overall, 50 patients were included in the study. A pathological complete response was achieved in 28.0% of patients. Considering second-order textural features, nine parameters showed a statistically significant difference between the two groups in ADC images, six parameters in PET images and four parameters in T2w images. Combining all the features selected for the three techniques in the same multivariate ROC curve analysis, we obtained an area under ROC curve of 0.863 (95% CI, 0.760–0.966), showing a sensitivity, specificity and accuracy at the Youden’s index of 100% (14/14), 64% (23/36) and 74% (37/50), respectively.

**Conclusion:**

PET/MRI texture analysis seems to represent a valuable tool in the identification of rectal cancer patients with a complete pathological response to nCRT.

## Introduction

Rectal cancer accounts for 736 000 new estimated cases and 340 000 estimated deaths in 2020 worldwide [1]. About 40% of rectal cancer cases are locally advanced (i.e. T3–T4) or node-positive at the time of diagnosis. Despite important advances in diagnosis and therapy, the treatment of these patients still represents a major oncological and surgical issue.

The standard of care for locally advanced rectal cancer (LARC) is currently represented by neoadjuvant chemoradiotherapy (nCRT) followed by total mesorectal excision (TME) [2,3]. Chemoradiotherapy alone achieves a pathological complete response (pCR) in about 20% of patients, who have been shown to have a better prognosis in terms of survival. In these cases, a conservative approach has been attempted, with radical surgery replaced by local excision (LE) or by strict clinical, radiologic and endoscopic follow-up alone, the so-called ‘Nonoperative Management’ [4,5].

Due to the drastic change in prognosis and to the possibility of new, noninvasive treatment approaches, it has become pivotal to identify reliable markers to predict the patient’s response to nCRT. Radiomics, which is the analysis of radiologic images based on the extraction of quantitative features, could represent a valid tool to predict response to therapy, and a good prognostic tool in the evaluation of rectal cancer patients, based on the assumption that genomics and proteomics characteristics should translate in macroscopic quantitative image features, which can be detected and analyzed using specific software packages. This kind of approach has already been applied to pelvic MRI, in order to assess the local extension of LARC, to predict prognosis in terms of survival and to foresee the patient’s response to treatment [6–13].

Some authors also explored the possibility to predict response to nCRT using both radiological (i.e. MRI) and nuclear medicine examination techniques (i.e. 18F-FDG PET/CT). Recently, Giannini *et al*. [14] analyzed 52 patients with LARC, identified as responders or nonresponders based on histologic findings, considering texture features from 18F-FDG PET-CT and MRI images. In this study, the model that combined PET and MRI radiomics features together proved to be far better than a model including only MRI features.

The use of PET/MRI has also been investigated. Some studies on the use of 18F-FDG PET/MRI for staging or restaging rectal cancer patients showed a slight advantage of this diagnostic technique over PET/CT in Tumor (T stage) and Nodal (N Stage) restaging [14–16].

Based on these premises, in the present study, we aimed to assess the ability of 18F-FDG PET/MRI to predict response to nCRT among patients with LARC undergoing curative-intent surgery at a third-level referral center.

## Materials and methods

### Patients’ selection

All patients undergoing curative-intent surgery after nCRT for LARC between 2015 and 2019 at a single-institution academic center were included in the study. The IRB of the institution approved the study. Individual informed consent was not required for the purposes of this study, and all the procedures followed were in accordance with the Helsinki Declaration. Patients older than 18 years, with histology-confirmed rectal cancer within 12 cm from the anal verge, who received nCRT, were restaged with 18F-FDG PET/MRI and underwent curative-intent surgery were included in the study. Patients with incomplete staging or restaging imaging and those who did not undergo PET/MRI were excluded.

Data on demographic, therapy-related and histopathological variables were collected. Specifically, patient demographic characteristics, including age and sex, were collected. Data regarding treatment details were also collected including the duration of chemoradiotherapy and the timing and type of surgical resection [i.e. LE; low anterior resection (LAR); and abdominoperineal resection (APR)]. Patients with a clinical complete or major response to neoadjuvant therapy were offered LE; in all other cases, a TME was performed, with surgery being scheduled 6–8 weeks after the accomplishment of nCRT.

Finally, data concerning final histopathological examination were obtained. Primary tumor regression was evaluated using the Mandard’s five-point assessment scheme: tumor regression grade (TRG) 1 complete regression with fibrosis and absence of residual cancer cells, TRG 2 presence or rare residual cancer cells, TRG 3 presence of residual tumor with predominantly fibrosis, TRG 4 residual cancer outgrowing fibrosis and TRG 5 no regressive change of the tumor [17].

Patients were classified into two groups, responders and nonresponders, based on TRG. Particularly, patients with TRG 1 were considered complete responders, whereas patients with TRG 2–5 were considered nonresponders.

### Imaging techniques

All included patients underwent PET/MRI scanning at restaging; a whole-body fully integrated PET/3-T MRI scanner (Biograph mMR, Siemens Healthcare, Germany) was adopted. Four- or five-bed positions were used, depending on the patients’ height, in order to obtain adequate coverage of the body from the vertex to the mid-tight. PET images were reconstructed using the ordered-subsets expectation-maximization algorithm and a Dixon VIBE MRI was used in order to generate an attenuation correction map [18]. Patients observed a 6-h fast before the examination, and 18F-FDG was administered intravenously at a dosage of 3 MBq/kg after checking blood glucose levels, with a maximum limit of 200 mg/dl. Image acquisition started 60 min after contrast administration, and the body was covered from vertex to mid-tights [19]. The acquisition protocol lasted 60 min. During the first 40 min, MRI sequences were acquired simultaneously with PET; the protocol included a whole-body axial diffusion-weighted imaging (DWI) sequences with a slice thickness of 5 mm, an echo time (TE) of 72 ms, a repetition time (TR) of 5100 ms and b-values 50–1000 s/mm^2^. Apparent diffusion coefficient (ADC) maps were consequently generated.

An additional 20 min protocol was dedicated to the examination of the pelvis. Particularly, we acquired bidimensional T2-weighted turbo spin-echo (T2w TSE) sequences of the pelvis in the sagittal plane, in the axial plane (i.e. perpendicular to the long axis of the tumor) and in the coronal plane (i.e. parallel to the long axis of the tumor); pelvic axial T2w TSE had a slice thickness of 3 mm, a TE of 123 ms and a TR of 4540 ms. During the 20 min acquisition of MRI, another bed position of PET covering the pelvis was recorded.

### Images analysis

**Fig. 1:**
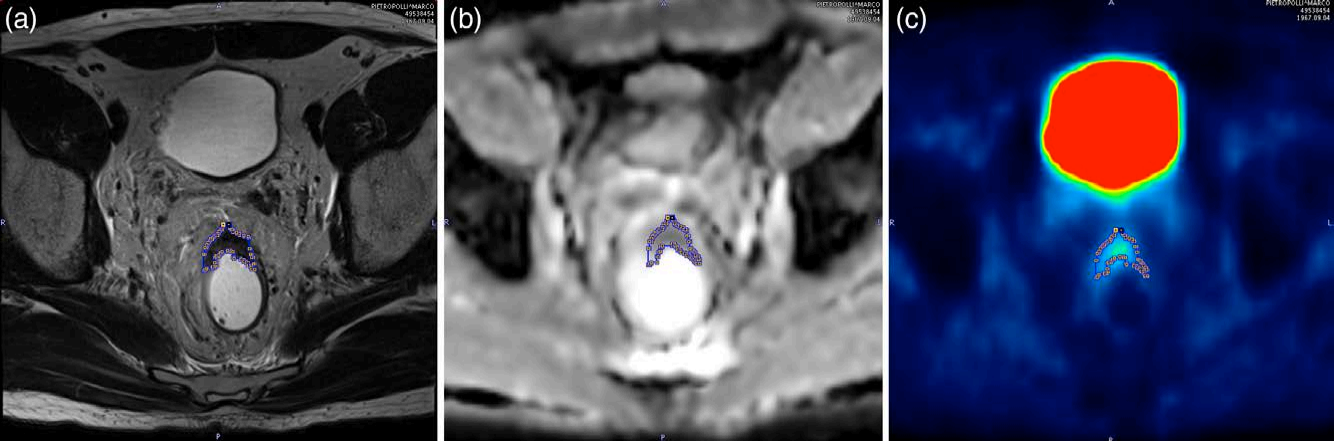
Manual region of interest (ROI) delineation with PMOD software, drawn along the boundaries of the rectal tumor in T2-weighted image (a) and then copied to the corresponding ADC map (b) and PET image (c).

For images analysis, we selected axial T2w TSE sequences, ADC maps and the 20 min PET acquisition of the pelvis. MR and PET images were analyzed on a dedicated workstation using the software PMOD (PMOD Technologies LLC, Zürich, Switzerland). PET images and ADC maps were reoriented and resliced in order to perfectly match to the axial T2w images. Two radiologists experienced in abdominal imaging (10 and 6 years, respectively), coming to a consensus in all patients, draw with the software a region of interest (ROI) around the boundaries of the lesion in each slice of the T2w images including the rectal tumor, obtaining a volume of interest (VOI). The ROIs obtained were then copied on the corresponding PET and ADC data sets (see Fig. 1). The dimensions of each voxel in the VOI were 0.75 × 0.75 × 3.00 mm; voxel-based standardized uptake value (SUV), ADC and T2w signal intensity values were extracted from the volume of the tumor.

SUV, ADC and T2w signal intensity values of each voxel inside the VOIs were automatically extracted by the software, which obtained 34 radiomic features from the image texture of each VOI for each dataset. The obtained radiomic features included six first-order parameters and 28 second-order gray-level cooccurrence matrix and second-order run-length matrix parameters. First-order statistics describe the distribution of pixels in the VOI using histograms, whereas second-order statistics describe how many neighbor pixels have the same gray level, and their relationship in the image.

### Statistical analysis

The radiomic features were extracted for each VOI in PET, ADC and T2w images and compared between responders (i.e. TRG 1 patients) and nonresponders (i.e. TRG 2–5 patients) using the Wilcoxon test and Bonferroni correction. The level of significance was taken as *P* < 0.01. A multivariable logistic regression model ROC-analysis was then performed using significant variables, in order to assess the accuracy of each imaging technique, and of all techniques combined, in identifying complete responders (i.e. TRG 1). The area under ROC curve (AUC), sensitivity, specificity and accuracy at the Youden’s index were then calculated. The statistical analyses were performed using the R software (R development core team, Vienna, Austria).

## Results

### Clinicopathological characteristics of the study group

**Fig. 2:**
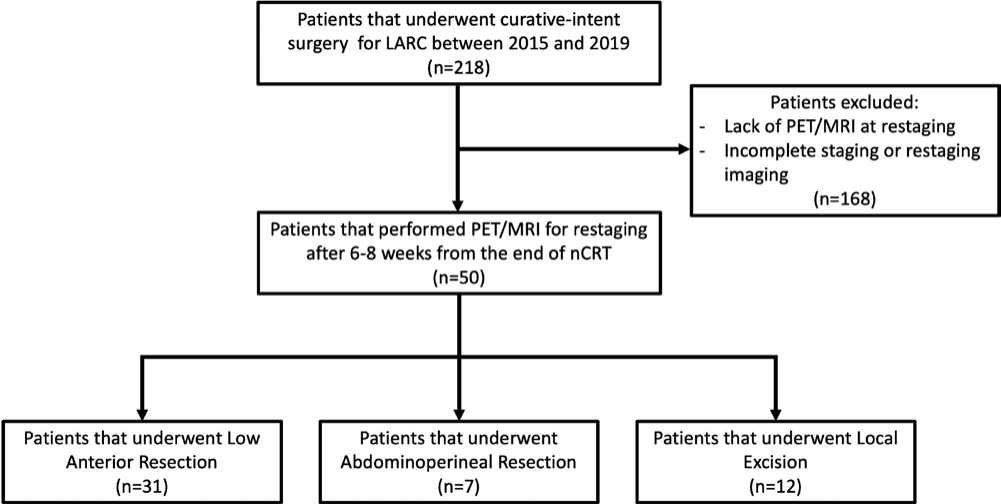
Flow-chart of the study.

Fifty patients, 36 males (72.0%) and 14 females (28.0%), were included in this study (see Table 1). The majority of patients were older than 60 years (*n* = 26; 52.0%). The mean duration of nCRT was 47.8 ± 20.9 days, and the meantime between nCRT and surgery was 76.7 ± 19.8 days. Overall, 38 patients (76.0%) underwent TME, whereas 12 patients (24.0%) underwent LE. Among patients undergoing TME, the large majority (31/38, 81.6%) underwent LAR, whereas seven out of 38 (18.4%) underwent an APR (see Fig. 2).

**Table 1:** Clinicopathological characteristics of the study group

	All	Responders (*n* = 14)	Nonresponders (*n* = 36)	*P*-value
Age (mean)	64.0 ± 9.4	61.5 ± 8.6	65.0 ± 9.7	0.24
Sex				
M	36	8	28	0.1473
F	14	6	8	0.1473
nCRT duration, days (mean)	47.8 ± 20.9	46.1 ± 14.0	48.5 ± 23.5	0.72
Time from nCRT to surgery, days (mean)	76.7 ± 19.8	84.9 ± 8.1	74.0 ± 21.8	0.0761
Type of intervention				
LE	12	8	4	0.0007
LAR	31	6	25	0.0852
APR	7	0	7	0.0782
ypT				
0	14	14	0	<0.0001
1	7	0	7	0.107
2	6	0	6	0.107
3	19	0	19	0.0006
4	4	0	4	0.198
ypN				
x	12	8	4	0.0007
0	22	5	17	0.4662
1	11	1	10	0.1174
2	5	0	5	0.1456

APR, abdominoperineal resection; LE, local excision; LAR, low anterior resection; nCRT, neoadjuvant chemoradiotherapy.

At final histopathological examination, 28.0% of patients achieved a pCR on the primary lesion (*n* = 18); 13 patients (26.0%) had histopathological T stage of 1–2, whereas the remaining had a T stage of 3–4 (*n* = 23; 46.0%). Sixteen patients (32.0%) were found to have positive lymph nodes on histopathologic examination. Overall, 14 patients were classified as responders based on TRG = 1 (28.0%), whereas the remaining 36 patients (72.0%) were classified as nonresponders. Noteworthy, one of the TRG1 patients showed locoregional lymph nodes metastases at histopathology.

### Volume of interests analysis

No first-order parameter showed statistically significant differences between responders and nonresponders in ADC, T2w and PET images analysis. When considering second-order textural features, nine parameters showed a statistically significant difference between the two groups in ADC images, six parameters in PET images and four parameters in T2w images (see Table 2).

**Table 2:** Second-order textural features analysis in ADC, T2w and PET images

Parameters	ADC	T2w	PET
Histogram mean	NS	NS	NS
Histogram variance	NS	NS	NS
Histogram skewness	NS	NS	NS
Histogram excess kurtosis	NS	NS	NS
Histogram energy	NS	NS	NS
Histogram entropy	NS	NS	NS
GLCM energy angular second moment uniformity	NS	NS	NS
GLCM contrast inertia variance	*P* = 0.0008	NS	NS
GLCM sum of squares variance	NS	NS	NS
GLCM homogeneity inverse different moment	*P* = 0.0005	*P* = 0.001	*P* = 0.0007
GLCM sum average	*P* = 0.001	*P* = 0.003	*P* = 0.0004
GLCM Sum variance	NS	NS	NS
GLCM Sum entropy	*P* = 0.0008	*P* = 0.002	*P* = 0.001
GLCM entropy	*P* = 0.0008	*P* = 0.002	NS
GLCM difference variance	NS	NS	NS
GLCM difference entropy	*P* = 0.0007	NS	*P* = 0.0007
GLCM information correlation	NS	NS	NS
GLCM autocorrelation	NS	NS	NS
GLCM dissimilarity	*P* = 0.0008	NS	NS
GLCM cluster shade	NS	NS	NS
GLCM cluster prominence	NS	NS	NS
GLCM maximum probability	NS	NS	NS
GLCM inverse difference	*P* = 0.0008	NS	NS
RLM short run emphasis	NS	NS	NS
RLM long run emphasis	NS	NS	*P* = 0.001
RLM low gray level emphasis	NS	NS	NS
RLM high gray level emphasis	NS	NS	*P* = 0.001
RLM gray level nonuniformity	NS	NS	NS
RLM run-length nonuniformity	NS	NS	NS
RLM run percentage	NS	NS	NS
RLM short run low gray-level emphasis	NS	NS	NS
RLM long run high gray-level emphasis	NS	NS	NS
RLM short run high gray-level emphasis	NS	NS	NS
RLM long run low gray-level emphasis	*P* = 0.0005	NS	NS

ADC, apparent diffusion coefficient; GLCM, gray-level cooccurrence matrix; RLM, run-length matrix; T2w, T2-weighted.

**Fig. 3:**
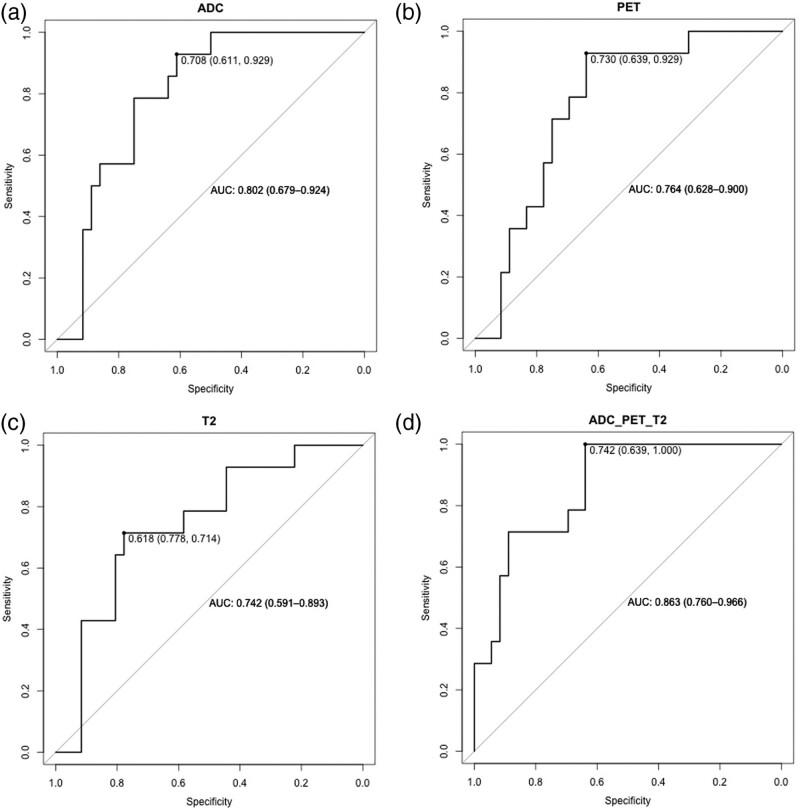
ROC curves for ADC (a), PET (b) and T2-weighted images (c) and multivariate ROC curve combining all the features selected for the three techniques (d).

When considering ADC, the ROC curve showed an AUC of 0.802 [95% confidence interval (CI), 0.679–0.924] and a sensitivity, specificity and accuracy at the Youden’s index of 93% (13/14), 61% (22/36) and 70% (35/50), respectively (see Fig. 3a).

In the PET ROC curve, an AUC of 0.764 (95% CI, 0.628–0.900) was detected, with a sensitivity, specificity and accuracy at the Youden’s index of 93% (13/14), 64% (23/36) and 72% (36/50), respectively (see Fig. 3b).

Finally, for T2w images, the ROC curve returned an AUC of 0.742 (95% CI, 0.591–0.893) with a sensitivity, specificity and accuracy at the Youden’s index of 71% (10/14), 78% (28/36) and 76% (38/50), respectively (see Fig. 3c).

When combining all the features selected for the three techniques (i.e. ADC, PET and T2w images) in the same multivariate ROC curve analysis, we obtained an AUC of 0.863 (95% CI, 0.760–0.966) showing a sensitivity, specificity and accuracy at the Youden’s index of 100% (14/14), 64% (23/36) and 74% (37/50), respectively (see Fig. 3d).

## Discussion

The treatment of LARC still represents an oncological and surgical challenge. With the introduction of nCRT, more patients with a diagnosis of LARC have been able to obtain a satisfying or even a complete regression of the primary tumor, leading to the possibility to undergo a more conservative surgical treatment, or even to avoid surgery [4]. LE has been proposed to spare such patients the morbidity of TME [20] while providing a histopathological assessment of the T stage [21]. Moreover, a nonoperative approach is currently offered to patients who achieve a clinical complete response (cCR) to nCRT in third referral centers, within specific research protocols [21,22]. In this setting, it has become pivotal to predict such patients’ response to nCRT, in order to offer a rectal sparing approach and to decrease the risk of complications of unnecessary surgical interventions.

However, the clinical definition of cCR still remains a matter of debate. According to the most widely accepted definition, digital rectal examination, proctoscopy, and pelvic MRI are required in order to identify complete responders [4,23,24]. Even so, up to 75% of patients considered to have a cCR show residual cancer at histopathological examination [25], and up to one-fourth develop local regrowth at 2 years [26]. Therefore, a more accurate tool for cCR identification is required.

Recently, different authors have reported the usefulness of the application of texture analysis to MR images, in order to predict pCR in such patients, generally with favorable results [10,12]. De Cecco *et al*. [10,11], in two different studies, identified kurtosis as a potential predictor of pCR in pretreatment and mid-treatment MRI. Moreover, Shu *et al*. [12] identified multiple T2w texture parameters in pre-nCRT and early-nCRT MRI scans, which showed significant differences between complete responders and partial or nonresponders, including variance, kurtosis, energy and entropy. Other authors also found significant correlations between texture parameters of MRI, including DWI sequences, and histopathologic results [27–33] or between MRI texture features and patients’ clinical outcomes [7,34]. Finally, correlations with tumor genetic mutations status [35–37] or lymph node metastatic involvement were reported [38].

PET/MRI has also been proposed as an effective imaging technique for the restaging of LARC following nCRT [39]. Giannini *et al*. [14] reported the results of texture analysis of PET/CT and MR images for predicting the complete response after nCRT in rectal cancer, showing a good accuracy (AUC, 0.86) for a combination of different textural features.

In our study, we considered patients undergoing restaging with PET/MRI following nCRT, and subsequent surgery with either TME or LE. Based on histopathological examination, patients were divided into responders (i.e. TRG 1) and nonresponders (i.e. TRG 2–5) to nCRT. Differently from other studies, we considered only the response to nCRT of the primary lesion and not of the locoregional metastatic lymph nodes (TRG vs. pCR). This choice was determined by the fact that we delineated for the texture analysis the primary lesion and, therefore, we considered more correct to perform a direct correlation with its histopathological data.

A logistic regression model containing nine second-order ADC, four second-order T2w and six second-order PET texture features was performed, obtaining an AUC of 0.863 (95% CI, 0.760–0.966). When considering five second-order PET texture parameters and one second-order MRI parameter, Giannini *et al*. [14] reported an AUC of 0.86. Thus, our results can be considered quite similar to those already reported. It is worth noticing that, in our study, the analysis was performed on coacquired PET and MR images thanks to the integrated PET/MRI [14].

The combined regression model, including T2w, ADC and PET images, yielded better results than the models including ADC, PET and T2w features alone, which showed for each technique an AUC of 0.742, 0.802 and 0.764, respectively.

This study has some points of strength, which should be considered. First of all, the number of included patients is reasonably high, when considering that PET/MRI has only been available for a few years in Italy and only in highly dedicated centers. Second, all acquisitions and analyses have been conducted by dedicated radiologist; all patients underwent a comparable nCRT regimen and were treated by a highly specialized surgical team. The pathologists performing histopathologic examinations were also dedicated to colorectal malignancies.

The main limitation of our study lies in its design. This is a retrospective, single-center study; thus, our results should be validated within a prospective, multicenter trial in order to be confirmed. Besides, although the number of patients can be considered to be high in the current clinical setting, it is still limited in order to draw definitive conclusions.

Moreover, even if a high number of radiomic features were obtained from the image texture of each VOI for each dataset (i.e. 19 second-order parameters) from three different techniques (T2w, ADC and PET), this could have increased the risk of overfitting [40,41].

Finally, PET/MRI represents an imaging technique, which is still not readily available in many hospitals and is not currently included in Italian guidelines on the management of rectal cancer [22].

### Conclusion

PET/MRI texture analysis seems to represent a valuable tool in the identification of rectal cancer patients with a complete pathological response to nCRT. If confirmed, our results could lead to an optimization of restaging techniques with the application of PET/MRI texture analysis, in the optics of a more tailored approach to the treatment of LARC.

## Acknowledgements

All the authors certify that they have no affiliations with or involvement in any organization or entity with any financial interest or nonfinancial interest in the subject matter or materials discussed in this manuscript.

### Conflicts of interest

There are no conflicts of interest.
